# Supplementary material for: The interactome of intact mitochondria by cross-linking mass spectrometry provides evidence for coexisting respiratory supercomplexes
Source: Mol Cell Proteomics. 2017 Dec 8;17(2):216–32. doi: 10.1074/mcp.RA117.000470 (PMC5795388; doi:10.1074/mcp.RA117.000470)
Supplement: Supplemental Data [file supp_RA117.000470_133922_0_supp_23978_fzffwf.zip › spectra_annotation/mito_DR_spectra_annotation/451-1-1-1-7-1.pdf]

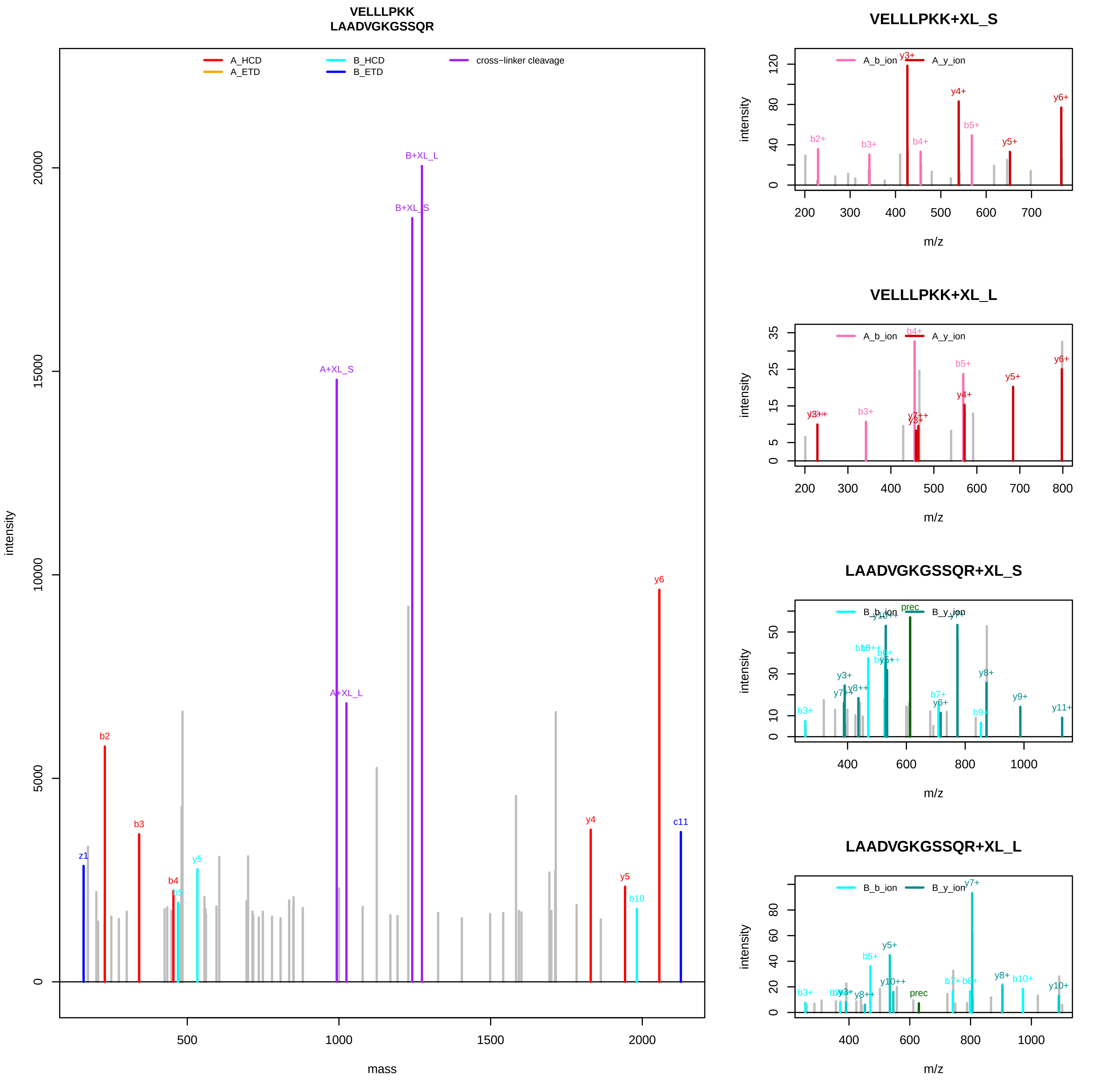

VELLLPKK
LAADVGKGSSQR
A_HCD
A_ETD
B_HCD
B_ETD
cross−linker cleavage
B+XL_L
B+XL_S
A+XL_S
A+XL_L
z1
b2
b3
b4
b5
y5
y4
y5
b10
y6
c11
20000
15000
10000
5000
0
intensity
500
1000
1500
2000
mass
VELLLPKK+XL_S
A_b_ion
A_y_ion
y3+
y4+
y6+
b2+
b3+
b4+
b5+
y5+
120
80
40
0
intensity
200
300
400
500
600
700
m/z
VELLLPKK+XL_L
A_b_ion
A_y_ion
b4+
b5+
y6+
y5+
y4+
b3+
35
25
15
5
0
intensity
200
300
400
500
600
700
800
m/z
LAADVGKGSSQR+XL_S
B_b_ion
B_y_ion
prec
y7+
y3+
y8++
b3+
b7+
y6+
y8+
y9+
y11+
50
30
10
0
intensity
400
600
800
1000
m/z
LAADVGKGSSQR+XL_L
B_b_ion
B_y_ion
y7+
y5+
b5+
y10++
b3+
y8++
prec
b7+
b8+
y8+
b10+
y10+
80
60
40
20
0
intensity
400
600
800
1000
m/z
